# Effects of lighting variability on locomotion in posterior cortical atrophy

**DOI:** 10.1002/trc2.12077

**Published:** 2020-10-07

**Authors:** Keir X. X. Yong, Ian D. McCarthy, Teresa Poole, Dilek Ocal, Ayako Suzuki, Tatsuto Suzuki, Kyriaki Mengoudi, Nikolaos Papadosifos, Derrick Boampong, Nick Tyler, Chris Frost, Sebastian J. Crutch

**Affiliations:** ^1^ Dementia Research Centre Department of Neurodegenerative Disease UCL Queen Square Institute of Neurology London UK; ^2^ Pedestrian Accessibility and Movement Environment Laboratory Department of Civil, Environmental and Geomatic Engineering Faculty of Engineering Science University College London London UK; ^3^ Department of Medical Statistics Faculty of Epidemiology and Population Health London School of Hygiene & Tropical Medicine London UK; ^4^ Centre for Medical Image Computing Department of Computer Science Faculty of Engineering Science University College London London UK

**Keywords:** Alzheimer's disease, dementia, locomotion, posterior cortical atrophy, visual perception

## Abstract

**Introduction:**

Clinical reports describe patients with Alzheimer's disease (AD) exhibiting atypical adaptive walking responses to the visual environment; however, there is limited empirical investigation of such behaviors or factors modulating their expression. We aim to evaluate effects of lighting‐based interventions and clinical presentation (visual‐ vs memory‐led) on walking function in participants with posterior cortical atrophy (PCA) and typical AD (tAD).

**Methods:**

Participants with PCA (n = 10), tAD (n = 9), and healthy controls (n = 12) walked to visible target destinations under different lighting conditions within two pilot repeated‐measures design investigations (Experiment 1: 32 trials per participant; Experiment 2: 36 trials per participant). Participants walked to destinations with the floorpath interrupted by shadows varying in spatial extent (Experiment 1: no, medium, high shadow) or with different localized parts of the environment illuminated (Experiment 2: target, middle, or distractor illuminated). The primary study outcome for both experimental tasks was completion time; secondary kinematic outcomes were proportions of steps identified as outliers (Experiment 1) and walking path directness (Experiment 2).

**Results:**

In Experiment 1, PCA participants overall demonstrated modest reductions in time taken to reach destinations when walking to destinations uninterrupted by shadows compared to high shadow conditions (7.1% reduction [95% confidence interval 2.5, 11.5; *P* = .003]). Experiment 2 found no evidence of differences in task performance for different localized lighting conditions in PCA participants overall. Neither experiment found evidence of differences in task performance between conditions in tAD or control participants overall. Completion time in both patient groups was longer relative to controls, and longer in PCA relative to tAD groups.

**Discussion:**

Findings represent a quantitative characterization of a clinical phenomenon involving patients misperceiving shadows, implicating dementia‐related cortico‐visual impairments. Results contribute to evidence‐based design guidelines for dementia‐friendly environments.

## 1 BACKGROUND

The visual environment may play an elevated role mediating everyday function in Alzheimer's disease (AD) and other dementia syndromes. This role is corroborated through clinical observations of patients overstepping perceptual variations in flooring, such as patterned carpeting or shadows, or being overly reliant on landmarks or conspicuous environmental features to support navigation.^1^, ^2^, ^3^, ^4^ Despite the promise of cost‐effective environmental adaptations facilitating patient autonomy and safety, repeated calls for high‐quality quantitative research in this area remain largely unmet,^2^, ^5^, ^6^ and existing studies have given limited consideration to patient clinical presentation.^7^

Posterior cortical atrophy (PCA) is the cardinal “visual dementia,” a neurodegenerative syndrome characterized by progressive cortico‐visual impairment and particular involvement of posterior parietal and occipito‐temporal regions.^8^, ^9^, ^10^, ^11^ PCA is most commonly caused by AD and is often considered the most common atypical AD presentation.^12^ PCA patients have relatively spared involvement of medial temporal regions, correspondingly demonstrating preserved episodic memory in early disease stages.^13^, ^14^ Cortico‐visual impairments arising in PCA, and to a lesser extent in typical, amnestic AD (tAD), include visuospatial and visuoperceptual deficits, diminished depth perception, and restrictions in the effective visual field.^9^, ^15^, ^16^, ^17^, ^18^ PCA is associated with environmental disorientation and has profound implications for independence, safety, and care considerations.^19^ At a relatively early stage, PCA patients may be unable to drive, read, dress independently, or reliably navigate familiar environments despite relatively preserved memory, language, and insight.^20^

Environmental adaptations have been recommended to support individuals living with dementia, particularly for PCA patients and AD patients with memory and visuospatial presentations.^19^, ^21^, ^22^ Recommendations based predominantly on professional guidance include strategic use of signage, contrast and salient visual features, patterned flooring, glare, and clutter.^2^, ^19^, ^23^, ^24^, ^25^ Lighting modulates contrast, perceived clutter, and visual saliency, factors particularly influencing object recognition and gaze location in PCA,^26^, ^27^ and lighting‐based interventions may promote functional outcomes and fall reductions in patients with all‐cause dementia.^23^, ^28^

Misperception of shadows has been reported in PCA and later‐stage AD^19^, ^24^ and is associated with hesitation or disorientation during navigation.^4^ Correspondingly, approaches to maintain uniformity of lighting and minimize shadows have been recommended in various forms.^23^, ^24^, ^29^ Use of localized lighting has been proposed to emphasize stairways, distinguish environmental settings, or act as orientation cues,^29^, ^30^ and location‐specific lighting to facilitate navigation has received limited support from one case study.^31^ However, previous reviews have noted generally low‐quality evidence regarding environmental design and patient function, contradictory recommendations for people with dementia and those with sight loss, and contentious guidance regarding appropriate lighting for people living with dementia.^2^, ^5^, ^23^

We evaluated effects of two lighting‐based interventions in participants with early‐stage PCA, tAD, and controls walking to visible destinations. We hypothesized that restricting shadows interrupting floorpaths to destinations would facilitate locomotion in mild PCA characterized by predominant cortico‐visual impairment, but not control or mild, predominantly amnestic tAD groups. We hypothesized that illuminating localized, task‐relevant parts of the environment (highlighting the intended destination rather than irrelevant environmental features) would facilitate locomotion in patient participants but not controls. Study outcomes were time taken to reach destinations, proportions of step times identified as outliers, and directness of routes to destinations.

## 2 METHODS

### 2.1 Participants

Ten PCA patients, 9 tAD patients, and 12 healthy controls were included. The number of PCA patients was limited owing to the low prevalence of PCA. PCA and tAD groups fulfilled clinical criteria for PCA‐pure^11^, ^32^, ^33^ and research criteria for probable AD,^34^ respectively. Groups were of comparable age, sex, and height, and patient groups were of comparable (mild) disease severity based on mean Mini‐Mental State Examination score (Table 1A). Molecular pathology was available for 5/10 PCA and 4/9 tAD patients; all were consistent with AD pathology. Ethical approval was provided by the National Research Ethics Service Committee London Queen Square; all participants provided written informed consent. A neuropsychological test battery was administered to PCA and tAD patients.

**TABLE 1:** Demographic information and neuropsychological scores of patients with PCA and tAD: (A) Demographic information and (B) neuropsychological raw scores and estimated performance relative to normative datasets of patients with PCA and tAD

*(A) Demographic information*		*PCA (N = 10)*	*tAD (N = 9)*	*Control (N = 12)* ^a^	
Sex (male:female)		8:2	7:2	8:4	
Age		69.5 (62.3, 75.3)	69.0 (62.0, 76.0)	70.5 (65.0, 72.3)	
Height (cm)		175.0 (170.2, 180.0)	175.0 (172.0, 178.0)	178.0 (169.8, 179.0)	
MMSE^b^ (/30)		24.5 (19.0, 28.8)	23.0 (21.0, 25.0)	—	
Amyloid beta PET/CSF consistent with AD^c^		5/5	4/4		

		*Raw Score*		*% patients below 5th%ile*	
*(B) Neuropsychology test*	*Max Score*	*PCA*	*tAD*	*PCA*	*tAD*
**Background neuropsychology**					
Short Recognition Memory Test for words (joint auditory/visual presentation)	25	20.5 (19.0, 23.3)	18.0 (16.0, 22.0)	10%	44%
Concrete Synonyms test	25	21.0 (21.0, 23.0)	22.5 (20.8, 23.3)	0%	11%
Naming (verbal description)	20	19.0 (19.0, 19.8)	17.0 (16.0, 19.0)	10%	33%
Calculation (GDA^d^)	24	6.5 (0, 10.0)	6.0 (2.0, 14.0)	40%	44%
Spelling (GDST^e^ Set B, first 20 items)	20	15.5 (8.0, 19.8)	14.5 (7.0, 20)	20%	11%
Gesture production test	15	13.5 (12.0, 15)	15 (15, 15)	—	—
Digit span (forward)	12	8.0 (6.3, 9.0)	8.0 (5.0, 10.0)	10%	22%
Max forward	8	6.5 (6.0, 7.0)	7.0 (5.0, 7.0)	—	‐
Digit span (backward)	12	3.5 (2.3, 5.8)	6.0 (4.0, 8.0)	0%	0%
Max backward	7	3.5 (2.3, 4.0)	4.0 (4.0, 5.0)	—	—
**Visual assessment**					
*Early visual processing*					
Visual acuity (CORVIST^f^): Snellen	6/9	6/9	6/9	—	—
Figure‐ground discrimination (VOSP^g^)	20	17.5 (16.3, 18.8)	19.0 (18.0, 19.0)	70%	33%
*Visuoperceptual processing*					
Fragmented letters (VOSP)	20	7.5 (1.3, 13.3)	19.0 (18.0, 20)	100%	11%^h^
*Visuospatial processing*					
Dot counting (VOSP)	10	5.0 (3.6, 6.8)	9.0 (8.0, 10)	90%	33%^h^
					
*Visual search*					
A Cancellation: Completion time	90s	65.0s (43.8, 90)	28.0s (23.0, 42.0)	80%	56%
A Cancellation: Number of letters missed	19	0.5 (0, 4.3)	0 (0, 0)	‐	‐

Medians and interquartile ranges are reported for demographic information and neuropsychological raw scores owing to non‐normal distribution of some variables.

Measures are adopted from a standard published cognitive battery.^26^, ^37^

Abbreviations: AD, Alzheimer's disease; CSF, cerebrospinal fluid; PCA, posterior cortical atrophy; PET, positron emission tomography; tAD, typical Alzheimer's disease.

^a^ Available cognitive measures (N = 7; Wechsler Abbreviated Scale of Intelligence, Recognition memory test for words, Paired Associate Learning^44^) and structural MRI (N = 8) did not provide evidence of cognitive impairment or neurodegeneration.

^b^ Mini‐Mental State Examination.

^c^ Positive amyloid scan on standard visual rating or CSF amyloid beta (Aβ)_1‐42_ ≤627 and/or tau/Aβ ratio > 0.52.

^d^ Graded Difficulty Arithmetic test.

^e^ Graded Difficulty Spelling Test.

^f^ Cortical Visual Screening Test.^42^

^g^ Visual Object and Space Perception Battery.^43^

^h^ Chi‐square tests indicate evidence of the proportion of patients performing below the 5^th^%ile differing between PCA and tAD groups (*P* ≤ .01).

### 2.2 Procedure

**FIGURE 1:**
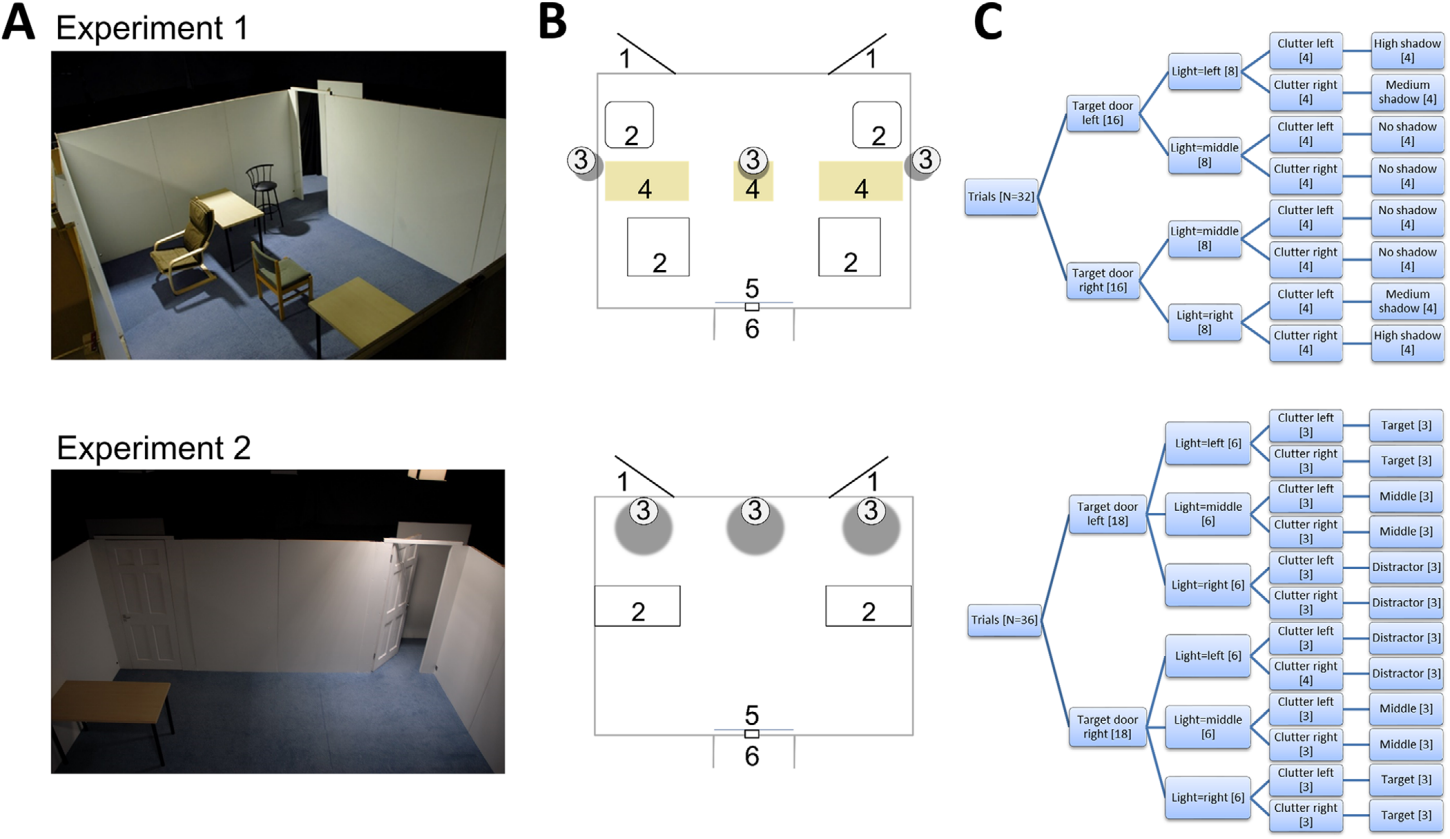
Experimental setting, schema, and room and lighting conditions for Experiments 1 and 2. A, Overhead view of the settings constructed at an accessibility laboratory (Pedestrian Accessibility Movement Environment Laboratory, University College London). B, Schematic of the setting; 1—target door positions, 2—moveable clutter positions (Experiment 1: chair; Experiment 2: table), 3—overhead lighting positions, 4—fixed furniture, 5—fixation point, 6—trial starting position. C, Room conditions and corresponding lighting conditions (Experiment 1: high shadow, medium shadow, no shadow; Experiment 2: target illuminated, middle illuminated, distractor illuminated); figures in square brackets indicate the number of trials per condition

The experimental setting was constructed at the Pedestrian Accessibility Movement and Environment Laboratory (PAMELA), University College London, which simulates real‐world settings while controlling for physical environmental conditions. The setting consisted of a room (main dimensions: 4.8 m[W] x 2 m[H] x 3.6 m[D]) with an entry corridor serving as the trial starting position (Figure 1A). Two doors (0.76 m[W] x 2 m[H]) were located 20° at 4.0 m from the starting position. For each trial, one door was opened at 46° to indicate the target destination. Participants walked to the target from a starting position. Between trials, a blind obscured the view of the room, with participants instructed to fixate on a fixed point positioned on the center of the blind at eye level. An Arduino‐based system fulfilled the following functions: signaling raising of the blind, recording trial start, and end time at 1000 Hz.

Two experiments were carried out; each was intended to explore effects of different lighting conditions on locomotion to visible destinations. Both experiments were of a repeated‐measures design ensuring an equal number of trials involving each of the following variables: door (left, right), lighting position (left, middle, right), and clutter position (left, right; Figure 1B). Experiment 1 additionally included fixed furniture. These designs resulted in different “room conditions”; room conditions produced three overall shadow/localized lighting conditions for each experiment:

- Experiment 1 (effects of shadow: 32 trials): eight room conditions produced the following conditions interrupting the floorpath to destinations with shadows varying in spatial extent: high shadow: eight trials, clutter on same side as target; medium shadow: eight trials, clutter on side opposite target; no shadow: eight trials, clutter on same side as target; eight trials, clutter on side opposite target (Figure 1C). Participants performed the task four times for each room condition. The no shadow condition comprised both of the two clutter/target combinations to match those combinations under medium and high shadow conditions.
- Experiment 2 (effects of localized lighting: 36 trials): 12 room conditions, producing the following conditions illuminating localized parts of the environment: target illuminated: 12 trials; middle illuminated: 12 trials; distractor illuminated: 12 trials. Participants performed the task three times for each room condition (Figure 1C).

Mean ground illuminance was matched between lighting conditions (Experiment 1: 17 lx; Experiment 2: 40 lx). Lighting position and clutter conditions were arranged in variants of a Latin square design (Experiment 1: N = 4; Experiment 2: N = 6). To control for order effects, variants were counterbalanced within participant and assigned randomly to participants, so that order varied between participants.

**HIGHLIGHTS**

- Perceptual variations in flooring may prompt atypical gait response in dementia.
- This phenomenon and its relation to clinical phenotype is little understood.
- Locomotion was assessed in posterior cortical atrophy (PCA) and typical Alzheimer's disease under three shadow conditions.
- Minimizing shadows was associated with faster and less hesitant locomotion in PCA.
- Cortico‐visual impairments are implicated in the expression of atypical gait.

**RESEARCH IN CONTEXT**

1. Systematic review: The authors reviewed the literature using traditional sources, meeting abstracts, and professional practice resources. Previous literature particularly emphasized appropriate lighting in promoting patient safety, functional independence, and quality of life. However, existing knowledge was predicated on observational study and professional guidance rather than empirical investigation, with limited consideration of clinical phenotype.
2. Interpretation: Findings provide evidence for effects of lighting variability on locomotion in patients with the posterior cortical atrophy (PCA) phenotype. Consistent with reports of misperceived shadows, hesitant gait response in PCA was associated with local lighting variations. Findings support practice recommendations to manage lighting variability, in addition to lighting level.
3. Future directions: the manuscript supports the contribution of cortico‐visual impairment toward atypical responses to the environment observed in dementia patients. Findings emphasize the need to consider clinical phenotype and environmental challenges in dementia care and the increasing field of in‐home monitoring through pervasive technology.

### 2.3 Study outcomes

Statistical analyses were performed using Stata (v. 14.1). For statistical tests we reported a two‐sided *P*‐value (alpha level: *P* < .05).

#### 2.3.1 Primary outcome: Completion time

Completion time was the difference between trial start and end times. End times were the first point when the target door threshold was crossed. Times were automatically detected for 1981/2090 (94.8%) trials using a Lightgate sensor; the remainder were manually determined owing to the Lightgate sensor not registering movement (total trials: 43 control, 48 PCA, 18 tAD). For each experiment, logarithmically (log) transformed completion times were analyzed using a mixed‐effects linear regression model, with fixed effects for group and room condition plus their interaction, and nested random effects for participant, and room condition within participant. Rather than fitting a standard repeated measures model (which would assume a homogeneous variance structure), we used a mixed model that allowed the random‐effects and within‐person residuals to have different variances in each group. A log transformation was used to improve the extent to which the normality assumptions made by the model were satisfied, with hypothesis tests carried out on this scale. A further advantage of this transformation is that comparisons between groups and room conditions can subsequently be expressed as percentage differences in geometric means after back transformation.

For each experiment, an overall between‐group comparison was made for log‐transformed mean completion times, averaged over all room conditions. The primary analyses of the shadow/lighting conditions estimated within group comparisons, the experiments being more highly statistically powered to detect these within‐participant effects than differences between groups. Within each group, linear combinations of the estimated mean completion times for each room condition provided estimated mean completion times for the three shadow/lighting conditions in each experiment (Experiment 1: high shadow, medium shadow, no shadow; Experiment 2: target illuminated, middle illuminated, distractor illuminated). A global Wald test assessed evidence for a difference across the three shadow/lighting conditions within each group. Only if statistically significant evidence was found of an overall difference across shadow/lighting conditions were pairwise comparisons between conditions carried out using Wald tests. No adjustments for multiple comparisons were made, but restricting the analysis to where the overall test was statistically significant reduces the chance of false positive findings. For Experiment 1, a global Wald test separately assessed whether there was evidence of an interaction between group and shadow condition. One participant (PCA) was unable to complete the last 18 trials for Experiment 2 owing to time constraints.

#### 2.3.2 Secondary outcomes

Wireless inertial measurement units (IMUs: Xsens MT) recorded feet kinematics at 75 Hz. For Experiment 1, IMU data were unavailable for all trials in one participant (PCA) and were unavailable for the last 16 trials for one participant (PCA) owing to depleted IMU battery, and for one trial for three participants (two PCA, one control). For Experiment 2, IMU data were unavailable for all trials in one participant (PCA), for the last 18 trials for the participant (PCA) owing to time constraints (as in Section 2.3.1), for two trials for one participant (control) and for one trial for two participants (one tAD, one control) owing to recording error.

##### Experiment 1: Step time outliers

Disproportionately long step times were considered to represent hesitations, consistent with previous observational^1^, ^4^ and gait investigations.^35^ Individual step times were calculated using threshold resultant acceleration values detecting when the foot was in contact with the floor. First and last step times were excluded for each trial. Medians of observed person‐specific median step times were calculated for each group, combining data from all trials irrespective of shadow condition. Outlying long step times were iteratively identified as follows.^35^ For each of the three groups, a three‐level linear mixed model was fitted including random effects for participant, “room condition within participant,” and “trial within participant and room condition.” Outliers with long step times were defined as observations with a standardized residual >3; these were removed, the model refitted, and outlier removal repeated until no further outliers were identified. Numbers of outliers and total numbers of steps per person under each shadow condition were displayed in bar charts, without formal statistical analysis.

##### Experiment 2: Walking path straightness index

Walking paths were estimated using dead reckoning. IMU accelerations were converted to standard coordinates and integrated to calculate velocity. Velocity drift was corrected based on periods when feet were in contact with the ground, and corrected velocity integrated to estimate foot position.^35^ Walking path straightness index (SI) was calculated as a ratio of the shortest possible path compared to the length of the path actually taken by a participant, with a range (0–1) for which 1 indicated maximum straightness.^36^ As SI is a proportion (bounded by 0 and 1) we used an empirical logit transformation, which makes the distributional assumption of normality more plausible by transforming the (0, 1) interval so that it is unbounded. A logit transformation improved the extent to which the normality assumptions made by the model were satisfied. Logit‐transformed SI values were analyzed using the same linear regression model used to analyze completion time (Section 2.3.1). Results on the logit‐transformed scale were back‐transformed to provide estimated average SI values.

## 3 RESULTS

Table 1B presents neuropsychological scores and estimated performance relative to normative datasets for PCA and tAD groups. Overall, tAD patients exhibited a predominantly amnesic syndrome, with a minority showing evidence of cortico‐visual impairments. In contrast, cortico‐visual impairments were evident in all PCA patients, particularly on visuoperceptual and visuospatial measures. Visual search performance was inefficient in both patient groups.

### 3.1 Experiment 1: Effects of shadow

**FIGURE 2:**
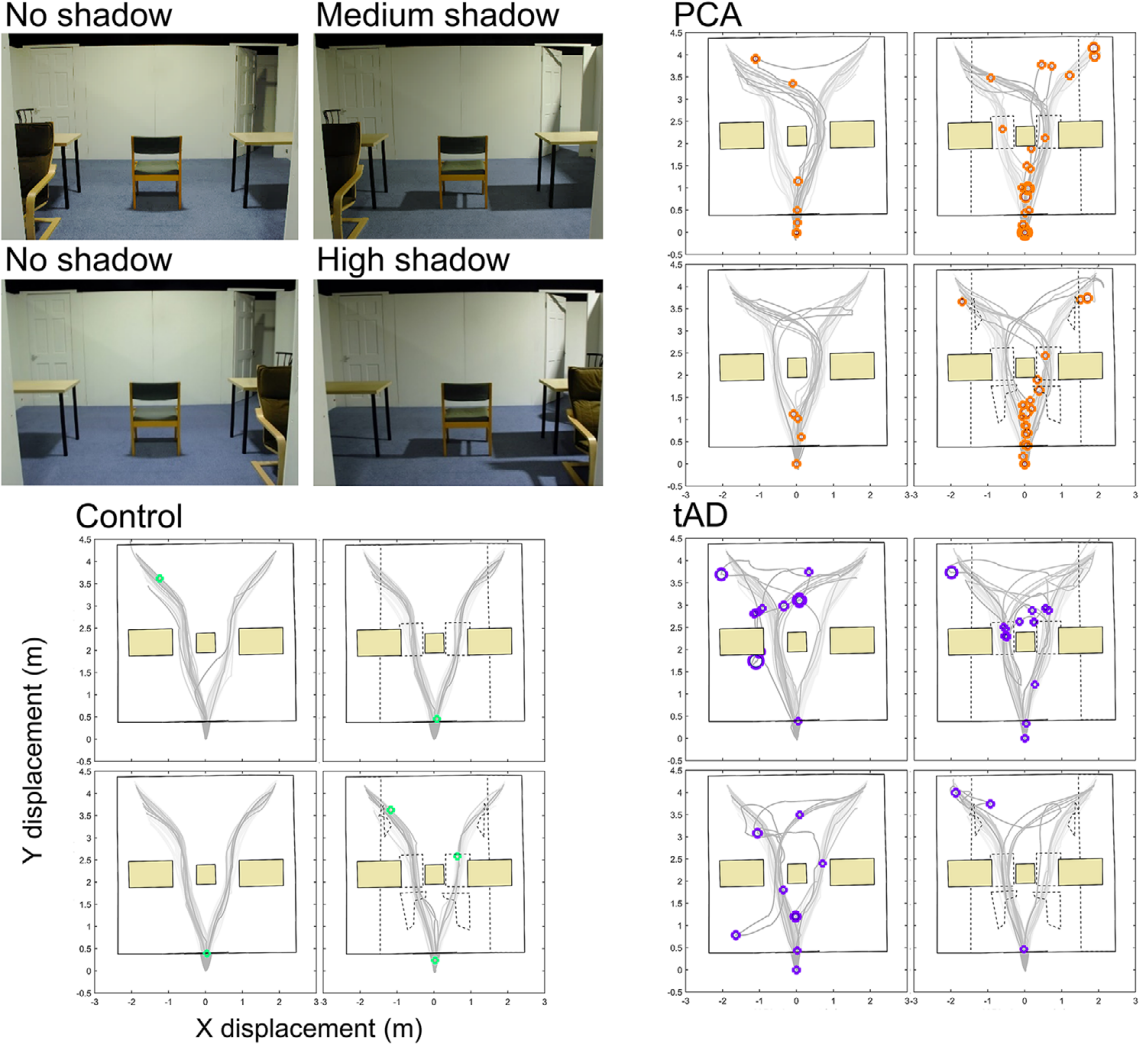
Experiment 1: step time outliers for posterior cortical atrophy, control, and typical Alzheimer's disease groups under shadow conditions. Step time outliers were determined using feet‐mounted inertial measurement units and overlaid over walking paths. Data are presented for combined door conditions (left/right). Marker size is proportionate to step time, shadows interrupting floorpaths to destination are indicated with dotted borders

**FIGURE 3:**
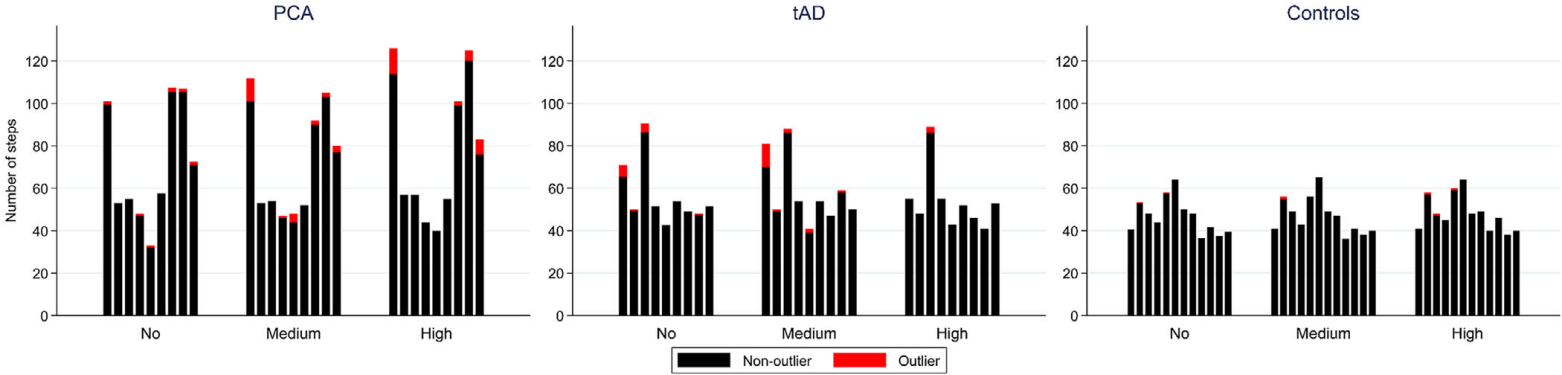
Experiment 1: Number of steps and step time outliers for posterior cortical atrophy, typical Alzheimer's disease, and control groups under shadow conditions. Number of steps per participant showing outlier step times. Number of steps and outliers are halved for no shadow results to take account of having double the number of trials compared to medium and high shadow. Within group participant results are presented in the same order for each shadow condition

Table 2 shows comparisons of estimated completion times for different shadow conditions in PCA, tAD, and control groups. Figures 2 and 3 show step time outliers for participant groups under different shadow conditions.

**TABLE 2:** Experiment 1: Effects of shadow on completion time

Primary outcome: Completion time							
	Geometric mean in seconds^a^ (95% CI)				Percentage reduction in completion time (95% CI)		
Group	*High Shadow*	*Medium Shadow*	*No Shadow*	Global test^b^	*Medium Shadow vs High*	*No Shadow vs High*	*No Shadow vs Medium*
PCA (N = 10)	10.4 (8.9, 12.3)	9.9 (8.4, 11.7)	9.7 (8.2, 11.4)	*P* = .01	4.9% (–0.6, 10.0)	7.1% (2.5, 11.5)	2.3% (–2.5, 6.9)
tAD (N = 9)	6.6 (5.9, 7.5)	6.7 (6.0, 7.6)	6.6 (5.9, 7.5)	*P* = .77	–2.1% (‐8.9, 4.2)	–0.3% (‐6.0, 5.1)	1.8% (–3.8, 7.1)
Controls (N = 12)	5.5 (5.1, 5.9)	5.5 (5.1, 5.9)	5.4 (5.1, 5.8)	*P* = .33	0.0% (–1.8, 1.7)	0.9% (–0.6, 2.4)	0.9% (–0.6, 2.4)

Estimated geometric means and percentage reduction in completion time results for PCA, tAD, and control groups.

Abbreviations: PCA, posterior cortical atrophy; tAD, typical Alzheimer's disease.

^a^ Geometric mean is the exponentiated mean of the estimated log transformed completion times.

^b^ Global test of the null hypothesis that within a participant group there is no difference between completion times under the three shadow conditions.

#### 3.1.1 Primary outcome: Completion time

Averaged over room conditions, overall task performance was slower in both the PCA (estimated geometric mean completion time: 9.9 seconds, 95% confidence interval [CI] 8.5, 11.7) and tAD groups (6.7 seconds, 95% CI 5.9, 7.5) relative to the control group (5.5 seconds, 95% CI 5.1, 5.9), and in the PCA relative to the tAD group. Between‐group differences were statistically significant (pairwise comparisons all *P* ≤ .004).

Within the PCA group, the global test found evidence (*P* = .01) for an overall difference between shadow conditions. This was largely driven by a statistically significant (*P* = .003) but relatively modest 7.1% (95% CI 2.5%, 11.5%) reduction in geometric mean completion times for the no versus high shadow condition comparison. Completion times for the medium shadow condition were intermediate, and not statistically significantly different from those for either the no (*P* = .34) or high shadow (*P* = .08) conditions (Table 2).

Analogously, global tests of geometric means comparing shadow conditions within the tAD and control groups were not statistically significant (*P* = .77 and *P* = .33, respectively); therefore, no pairwise comparisons were carried out. Despite the differences between the participant groups in the statistical significance of the shadow effect comparisons, a global joint test found no evidence that shadow effects differed overall between participant groups (*P* = .15), although it should be noted that this test is not as highly statistically powered as those investigating effects within individual groups.

#### 3.1.2 Secondary outcome: Step time outliers

Qualitatively, an increased proportion of outliers was observed as participants approached the shadow regions for the PCA group relative to tAD and control groups (Figure 2; Video in supporting information). Combining data from all trials irrespective of shadow condition, medians of observed person‐specific median step times were 0.71 seconds in PCA (interquartile range [IQR; 0.67, 0.72]), 0.63 seconds in tAD (IQR [0.57, 0.66]), and 0.58 seconds in controls (IQR [0.54, 0.59]).

Figure 3 displays the total number of steps taken by each participant in each shadow condition, with the number of step time outliers shown in red. Because there were 16 no shadow condition trials and only 8 medium and 8 high shadow condition trials per participant, numbers of steps and step time outliers in the no shadow condition are halved to aid visual comparability. Overall, controls typically took fewer steps than both patient groups, with a total of only six step time outliers across all participants and shadow conditions. There was marked variability in the numbers of steps, and numbers of outliers, in both patient groups, with some patients having few or no outliers. Typically those patients who had outliers (considered hesitant steps) had more when there were shadows, but this pattern was not universal: one tAD patient had multiple outliers in the no and medium shadow conditions, but none under high shadow. The small numbers of participants, coupled with the heterogeneity in the results seen, meant that no formal statistical analysis is reported.

### 3.2 Experiment 2: Effects of localized lighting

Table 3 shows comparisons of estimated completion times and walking path SI for different localized lighting conditions in PCA, tAD, and control groups. Figure S1 in supporting information shows walking paths for participant groups under different localized lighting conditions.

**TABLE 3:** Experiment 2: Effects of localized lighting on completion time and walking path straightness index (SI): (A) Estimated geometric means and percentage reduction in completion time results; (B) estimated walking path SI for PCA, tAD, and control groups

(A) Primary outcome: Completion time							(B) Secondary outcome: walking path straightness index (SI)			
	Geometric mean^a^ in seconds (95% CI)				Percentage reduction in completion time (95% CI)		Estimated average SI (95% CI)			
Group	*Target*	*Middle*	*Distractor*	Global test^b^	*Target vs Distractor*	*Middle vs Distractor*	*Target*	*Middle*	*Distractor*	Global test^c^
PCA (N = 10^‡^)	9.0 (7.5, 10.7)	9.0 (7.5, 10.8)	9.3 (7.8, 11.2)	*P* = .21	4.0% (−0.7, 8.4)	3.2% (−1.5, 7.7)	0.96 (0.95, 0.97)	0.96 (0.95, 0.96)	0.95 (0.94, 0.96)	*P* = .20
tAD (N = 9)	6.5 (5.7, 7.4)	6.6 (5.8, 7.5)	6.7 (5.9, 7.7)	*P* = .61	3.2% (−3.5, 9.4)	2.5% (−4.3, 8.8)	0.97 (0.96, 0.98)	0.97 (0.96, 0.98)	0.97 (0.96, 0.98)	*P* = .81
Controls (N = 12)	5.4 (5.0, 5.7)	5.3 (5.0, 5.7)	5.3 (5.0, 5.7)	*P* = .75	−0.4% (‐2.1, 1.3)	0.2% (‐1.5, 1.9)	0.97 (0.97, 0.98)	0.97 (0.97, 0.98)	0.97 (0.97, 0.98)	*P* = .93

Abbreviations: PCA, posterior cortical atrophy; SI, straightness index; tAD, typical Alzheimer's disease.

^a^ Geometric mean is the exponentiated mean of the estimated log transformed completion times.

^b^ Global test of the null hypothesis that within a participant group there is no difference between completion times under the three localized lighting conditions.

^c^ Walking path SI data were unavailable for one PCA participant owing to recording error, so N = 9 for SI analyses.

#### 3.2.1 Primary outcome: Completion time

Averaged over room conditions, overall task performance was slower in both PCA (estimated geometric mean completion time: 9.1 seconds, 95% CI 7.6, 10.9) and tAD groups (6.6 seconds, 95% CI 5.8, 7.5) relative to the control group (5.3 seconds, 95% CI 5.0, 5.7), and in the PCA relative to the tAD group. Between‐group differences were statistically significant (pairwise comparisons all *P* ≤ .003).

Global tests found no evidence within any group for a difference in completion times between localized lighting conditions (PCA: *P* = .21; tAD: *P* = .91; controls: *P* = .76).

#### 3.2.2 Secondary outcome: Walking path straightness index

Medians of observed person‐specific median SI were slightly lower in the PCA (0.95, IQR [0.94, 0.96]) relative to the tAD (0.97, IQR [0.97, 0.98]) and control groups (0.97, IQR [0.97, 0.97]), with observed data qualitatively suggesting a tendency toward some patients taking indirect routes to destinations (Figure S1).

Global tests found no evidence within any group for differences in SI between localized lighting conditions (Table 3B).

## 4 DISCUSSION

The current pilot investigation presents empirical evidence of lighting‐based interventions on functional locomotion to visible destinations. Two tasks assessed effects of lighting variability, while controlling the overall level of ground illuminance. For both tasks, patient groups took longer to reach target destinations than controls. Qualitatively, some patients displayed a tendency to take indirect and/or hesitant routes to destinations as determined through body kinematic analysis. Cortico‐visual impairments were notably apparent in the PCA group, the only group to show any evidence of effects of lighting interventions on task performance.

To perform ecological assessment of a complex behavior, a repeated‐measures, randomized, and counterbalanced design and detailed kinematic and neuropsychological analyses were used. Findings support previous proposals of atypical walking adaptation arising from AD‐related cortico‐visual impairments, and contribute to recent evidence on environmental adaptations facilitating functional locomotion in neurodegenerative disease.^37^

The first experiment investigated effects of shadows interrupting floorpaths to destinations. In PCA, but not tAD or control groups, there was evidence for reduced completion time when limiting shadows interrupting the path. This shadow effect was largely driven by a statistically significant but modest reduction in geometric mean completion time for no compared to high shadow conditions; geometric mean completion time for the medium shadow condition was intermediate between these two.

There was considerable variability in the secondary outcome (step time outliers–considered hesitant steps) in both patient groups. A number of patients had no hesitations; others took substantially more steps with more outliers relative to controls. While there was an overall tendency for patients to exhibit more outliers when shadows interrupted floorpaths to destinations, the extent of heterogeneity and number of patients who did not exhibit outliers precluded formal assessment of differences between shadow conditions. Qualitative analysis of detected outliers suggested differing spatial profiles in the occurrence of hesitant steps between patient groups, likely relating to the principal differences in group presentation (visual versus memory‐led). Taken altogether, results provide evidence for modest benefits of minimizing shadows on patient function in the PCA group, though findings may be relevant to a proportion of tAD patients exhibiting cortico‐visual impairment.^17^, ^38^

The second experiment investigated effects of localized lighting of task‐relevant (highlighting the intended destination) versus irrelevant environmental features. There were no statistically significant improvements in any group on primary (completion time) and secondary (walking path directness) outcome measures when highlighting destinations relative to an irrelevant “distractor” destination. The lack of evidence supporting beneficial effects of localized lighting on patient locomotion meant that we were unable to reject the null hypotheses for this experimental task: that patient task performance does not differ between localized lighting conditions and that there would be no difference in the effect of localized lighting between patient and control groups. Findings did not provide evidence to support previous suggestions of localized lighting assisting orientation and navigation,^24^, ^30^, ^31^ at least within this controlled and relatively simple environment.

Strengths of the current investigation include patient participants well characterized in neuropsychological phenotype, sensitive kinematic measures, a high number of trials per participant, and a randomized and counterbalanced design to control for order effects. Room conditions were balanced and mean ground illuminance matched between condition. The step time outlier detection method located disproportionately long step times for individuals during functional walking to destinations. This method does not require a gait laboratory; future work might investigate constrained gait tasks, community mobility, and feasibility of evaluating effects of environmental adaptations on gait variability and falls.^39^

Limitations of this pilot study include the presentation of the clutter variable being affected by overhead lighting position; this interrelation precluded independent analysis of lighting and clutter position. To better meet assumptions underlying statistical methods, completion time and straightness index outcome measures were log‐ and logit‐transformed, respectively. While hypothesis testing conducted on raw versus transformed data is not necessarily equivalent,^40^ validity of estimates and hypothesis tests depends on model assumptions being met. While the mild PCA patient group is of comparable size to interventional studies of atypical clinical phenotypes closely associated with AD (eg, logopenic variant^41^), the current pilot investigation involved a relatively small number of mostly young‐onset patients; statistical tests and confidence intervals should be interpreted with caution, with findings requiring replication and validation in larger samples. Furthermore, while molecular or pathophysiological evidence of AD was available in half of patients, non‐AD pathologies cannot definitively be ruled out. Factors limiting generalizability of findings include the unfamiliar experimental setting to control for familiarity and environmental conditions.

The current study provides evidence of lighting‐induced perceptual changes to the physical environment (restricting shadows) supporting walking performance in PCA, but not tAD or control groups. Future work might investigate other syndromes (corticobasal, dysexecutive) and sensory conditions to further explore the interaction between clinical phenotype and environment. Findings may contribute to compensatory approaches to maximize function in PCA, and encourage consideration of environmental conditions in the expanding field of dementia and pervasive health care.

## CONFLICTS OF INTEREST

The authors have no conflicts of interest to declare.

## Supporting information

Supporting Information.

Supporting Information.
