# Supplementary material for: Effects of lighting variability on locomotion in posterior cortical atrophy
Source: Alzheimers Dement (N Y). 2020 Oct 7;6(1):e12077. doi: 10.1002/trc2.12077 (PMC7539669; doi:10.1002/trc2.12077)
Supplement: Supplementary file 2 — Supporting Information. [file TRC2-6-e12077-s002.docx]

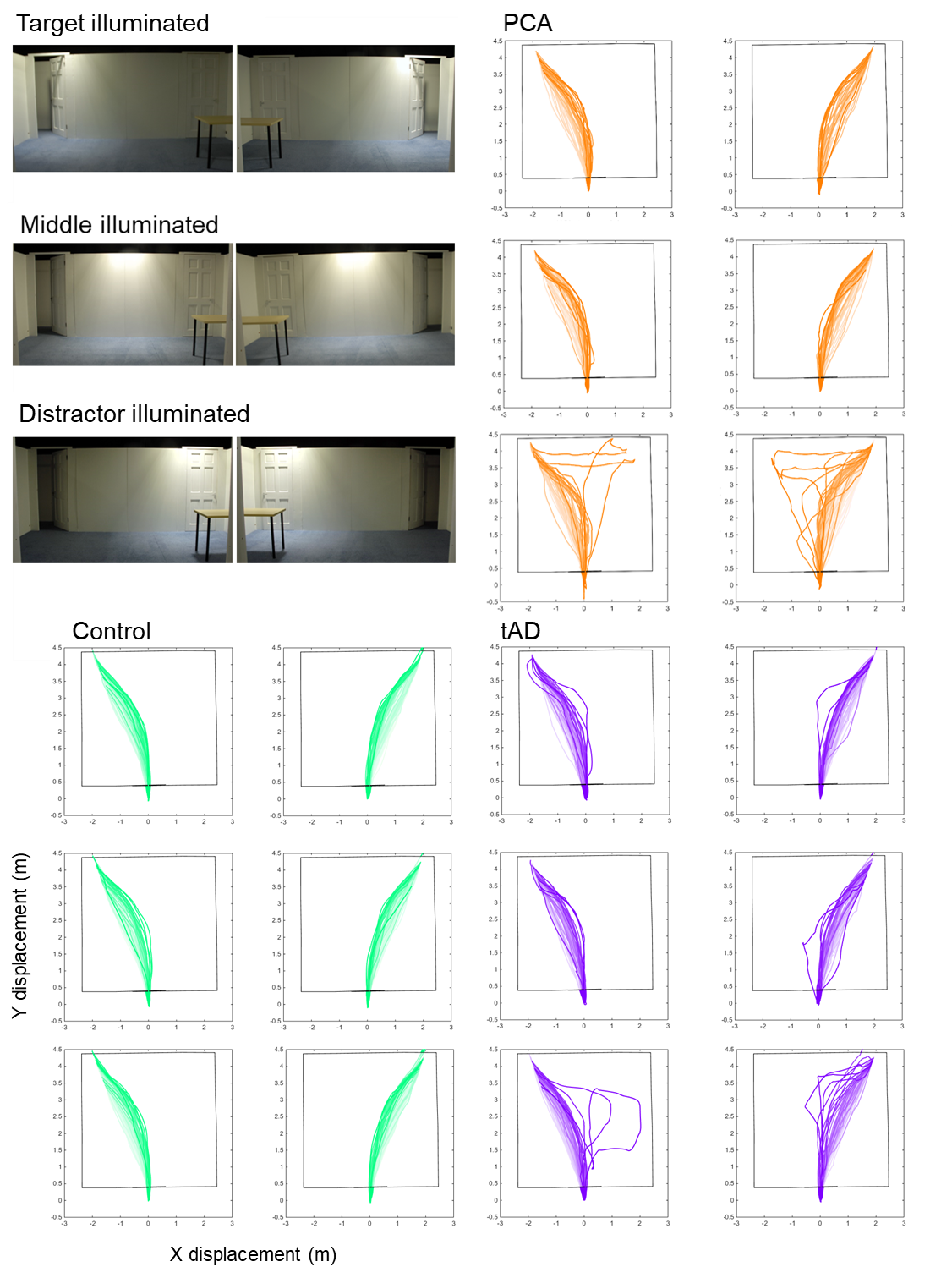
**Supplementary Figure. Experiment 2: walking paths for the PCA group under localized lighting conditions.** Walking paths are determined using feet-mounted inertial measurement units and the dead reckoning technique. Walking path data are presented for separate door conditions (left/right) across both clutter position conditions (left/right).
